# Association of Visitor Contact Precautions With Estimated Hospital-Onset *Clostridioides difficile* Infection Rates in Acute Care Hospitals

**DOI:** 10.1001/jamanetworkopen.2021.0361

**Published:** 2021-02-26

**Authors:** Elizabeth Scaria, Anna K. Barker, Oguzhan Alagoz, Nasia Safdar

**Affiliations:** 1 Department of Industrial and Systems Engineering, University of Wisconsin–Madison, Madison; 2 Department of Internal Medicine, University of Michigan, Ann Arbor; 3 Population Health Sciences, School of Medicine and Public Health, University of Wisconsin–Madison, Madison; 4 Division of Infectious Diseases, Department of Medicine, School of Medicine and Public Health, University of Wisconsin–Madison, Madison; 5 William S. Middleton Memorial Veterans Hospital, Madison, Wisconsin

## Abstract

**Importance:**

Visitor contact precautions (VCPs) are commonly used to reduce the transmission of *Clostridioides difficile* at health care institutions. Implementing VCPs requires considerable personnel and personal protective equipment resources. However, it is unknown whether VCPs are associated with reduced hospital-onset *C difficile* infection (HO-CDI) rates.

**Objective:**

To estimate the association between VCPs and HO-CDI rates using simulation modeling.

**Design, Setting, and Participants:**

This simulation study, conducted between July 27, 2020, and August 11, 2020, used an established agent-based simulation model of *C difficile* transmission in a 200-bed acute care adult hospital to estimate the association between VCPs and HO-CDI while varying assumptions about factors such as patient susceptibility, behavior, and *C difficile* transmission. The model simulated hospital activity for 1 year among a homogeneous, simulated adult population.

**Interventions:**

No VCP use vs ideal use of VCPs under different hospital configurations.

**Main Outcomes and Measures:**

The rate of HO-CDI per 10 000 patient-days according to the Centers for Disease Control and Prevention’s definition of HO-CDI.

**Results:**

With use of the simulation model, the baseline rate of HO-CDI was 7.94 10 000 patient-days (95% CI, 7.91-7.98 per 10 000 patient-days) with no VCP use compared with 7.97 per 10 000 patient-days (95% CI, 7.93-8.01 per 10 000 patient-days) with ideal VCP use. Visitor contact precautions were not associated with a reduction of more than 1% in HO-CDI rates in any of the tested scenarios and hospital settings. Independently increasing the hand-hygiene compliance of the average health care worker and environmental cleaning compliance by no more than 2% each was associated with greater HO-CDI reduction compared with all other scenarios, including VCPs.

**Conclusions and Relevance:**

In this simulation study, the association between VCPs and HO-CDI was minimal, but improvements in health care worker hand hygiene and environmental cleaning were associated with greater reductions in estimated HO-CDI. Hospitals may achieve a higher rate of reduction for HO-CDI by focusing on making small improvements in compliance with interventions other than VCP.

## Key Points

**Question:**

Is there an association between visitor contact precautions and hospital-onset *Clostridioides difficile* infection in acute care hospitals?

**Findings:**

In this simulation study of a generic acute care adult hospital, in all experiments, use of visitor contact precautions was associated with a decrease of 1% or less in the rate of hospital-onset *C difficile* infection; small improvements in hand hygiene and environmental cleaning were associated with larger decreases in hospital-onset *C difficile* infection.

**Meaning:**

In this study, visitor contact precautions were associated with minimal reductions in hospital-onset *C difficile* infections, suggesting that hospitals may achieve a larger reduction through other interventions.

## Teaser

This simulation study estimates the association between visitor contact precautions and the rate of hospital-onset *C difficile* infection in an acute care hospital.

## Introduction

*Clostridioides difficile* infection (CDI) is a major infection associated with health care, and its containment is considered a public health priority.^1^ In health care settings, *C difficile* transmission is believed to occur primarily through the physical interactions among patients, health care workers, visitors, and the environment. Recent guidelines from leading infectious diseases societies recommend several infection control interventions to curtail *C difficile* transmission.^2,3^ Thus, many institutions have developed CDI prevention bundles, which incorporate multiple intervention strategies simultaneously. Implementing complex bundles with high fidelity requires extensive resources.^4^ However, infection control programs are markedly underfunded at hospitals, with only half of hospitals budgeting money specifically for an infection control department.^5^ In the setting of known financial constraints, it is crucial to identify and prioritize the most promising interventions to reduce hospital-onset CDI (HO-CDI).

Prevention bundles for CDI typically include a visitor contact precautions (VCPs) component, which requires visitors to don gowns and gloves when entering the room of a patient with CDI. In a recent survey of 245 North American hospitals, 71% offered education materials to visitors about contact precautions for *C difficile* and 82% had specific contact-precaution policies for visitors of patients with CDI.^3^ The 2014 infectious diseases guidelines consider the use of contact precautions for visitors who enter the room of a patient with CDI as an unresolved issue without substantial supporting evidence; infection control guidelines recommend the use of VCPs for patients with CDI but also note a lack of supporting evidence.^3,6^ The 2017 infectious diseases guidelines specifically recommend contact precautions for health care workers.^2^

Using contact precautions for patients with CDI when they are cared for by health care workers is essential to reduce transmission from patient to patient and from patient to health care worker. However, visitors, unlike health care workers, do not interact with multiple patients and do not usually participate in the physical care of the patient. Thus, the benefit of VCPs is unclear. Moreover, VCPs may be associated with adverse effects for patients if their visitors are required to don gowns and gloves before interacting with them. Unlike many other infection control interventions, in addition to the cost and resource implications, there may be an association between quality of life and contact precautions.^7^ Some studies have reported increased delirium and depression in patients for whom contact precautions are used; other studies have not found such an association.^7,8,9,10^ It is possible that adverse effects associated with contact precautions are increased by VCPs because isolating patients from their visiting family and friends could be distressing. Receiving visitors has been shown to be associated with greater satisfaction with health care among patients, but patients under contact precautions may receive a smaller number of visitors.^11,12^ Any potential benefits associated with VCPs must be considered in the context of detriments in patient outcomes. The risks and costs associated with VCPs in typical conditions may outweigh the benefits for 2 other health care–associated infections: methicillin-resistant *Staphylococcus aureus* (MRSA) and vancomycin-resistant *Enterococcus* (VRE) infections. Recent guidelines recommend against VCPs for endemic MRSA and VRE infections because these infections are prevalent in the community.^3^

Data on the association of VCPs with CDI transmission are essential to guide decision-making regarding this intervention. To our knowledge, no study has estimated the association of VCPs with hospital-onset CDI. The purpose of this study was to use simulation modeling to estimate the association between VCPs and HO-CDI. Estimating this association was challenging because it may differ according to hospital and community characteristics. For example, there is likely a differential association of VCPs with the rate of HO-CDI in a hospital where most patients are susceptible to CDI compared with a hospital where a small proportion of the patients are susceptible. Simulation modeling is inherently able to test large numbers of configurations, and as such, it is a useful alternative to conventional epidemiologic studies in this context.

## Methods

### Overview of the Agent-Based Simulation Model

**Figure 1.**
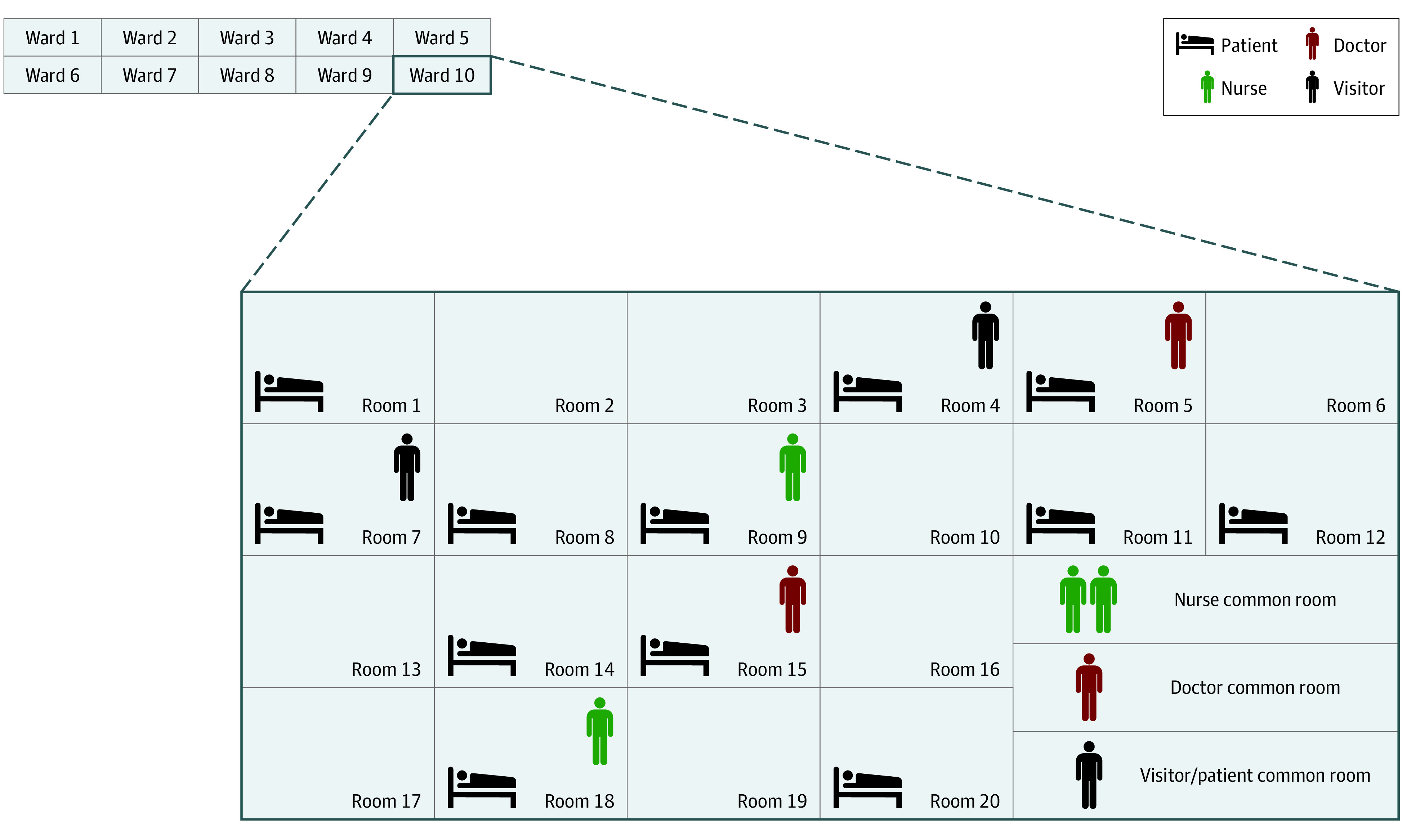
Layout of the Agent-Based Model Agent-based simulation model of *Clostridiodides difficile* transmission in an acute care, tertiary adult hospital. This model replicated events associated with *C difficile* infection in a 200-bed dynamic hospital environment representing an average-sized US adult hospital and included 4 types of individuals as agents: patients, visitors, nurses, and physicians.

This simulation study was conducted between July 27, 2020, and August 11, 2020, using a previously developed agent-based simulation model of *C difficile* transmission in an acute care, tertiary adult hospital.^4^ This model replicated events associated with CDI in a 200-bed dynamic hospital environment representing an average-sized US adult hospital and included 4 types of individuals as agents: patients, visitors, nurses, and physicians (Figure 1).^4^ The University of Wisconsin Health Sciences Institutional Review Board approved the study and waived the need for informed consent because the data were deidentified. The study followed the Consolidated Health Economic Evaluation Reporting Standards (CHEERS) reporting guideline.

The model represented the CDI status of the patients using 1 of 9 possible disease states. The model updated the clinical state of each patient in the hospital every 6 hours using a discrete-time Markov chain structure (eTable 1 and the eFigure in the Supplement). Patients in the colonized, infected, recolonized, or recurrent infection states can transmit *C difficile* to other agents and the environment through interactions with them. Visitors, nurses, and physicians who are contaminated with *C difficile* from patients or the environment can in turn transmit *C difficile* to other susceptible patients and the environment. Similarly, any agent interacting with a contaminated environment can become exposed to *C difficile*. The probability of transmission for each interaction depends on the agent types involved and the duration of the interaction. The key parameters associated with *C difficile* transmission and other model inputs are presented in eTable 2 in the Supplement.

The initial model was developed using NetLogo software, version 5.3.1 (Center for Connected Learning and Computer-Based Modeling, Northwestern University) and subsequently translated into the Java programming language (Oracle Corp) for faster speeds and flexibility as described in the eAppendix and eTables 3-7 in the Supplement.^4^ All experiments were conducted using the Java version of the model. The original NetLogo model was validated through face validation, parameter sensitivity analyses, and cross-validation, which involved comparing model outputs with those from *C difficile* infection–control studies in the literature. Both versions used common random numbers generated by the Colt Project’s Mersenne Twister algorithm to reduce variation and to directly compare runs under different intervention scenarios.^4,13^ A detailed description of the model is provided elsewhere.^4,14^

### Visitor Agent Logic

**Figure 2.**
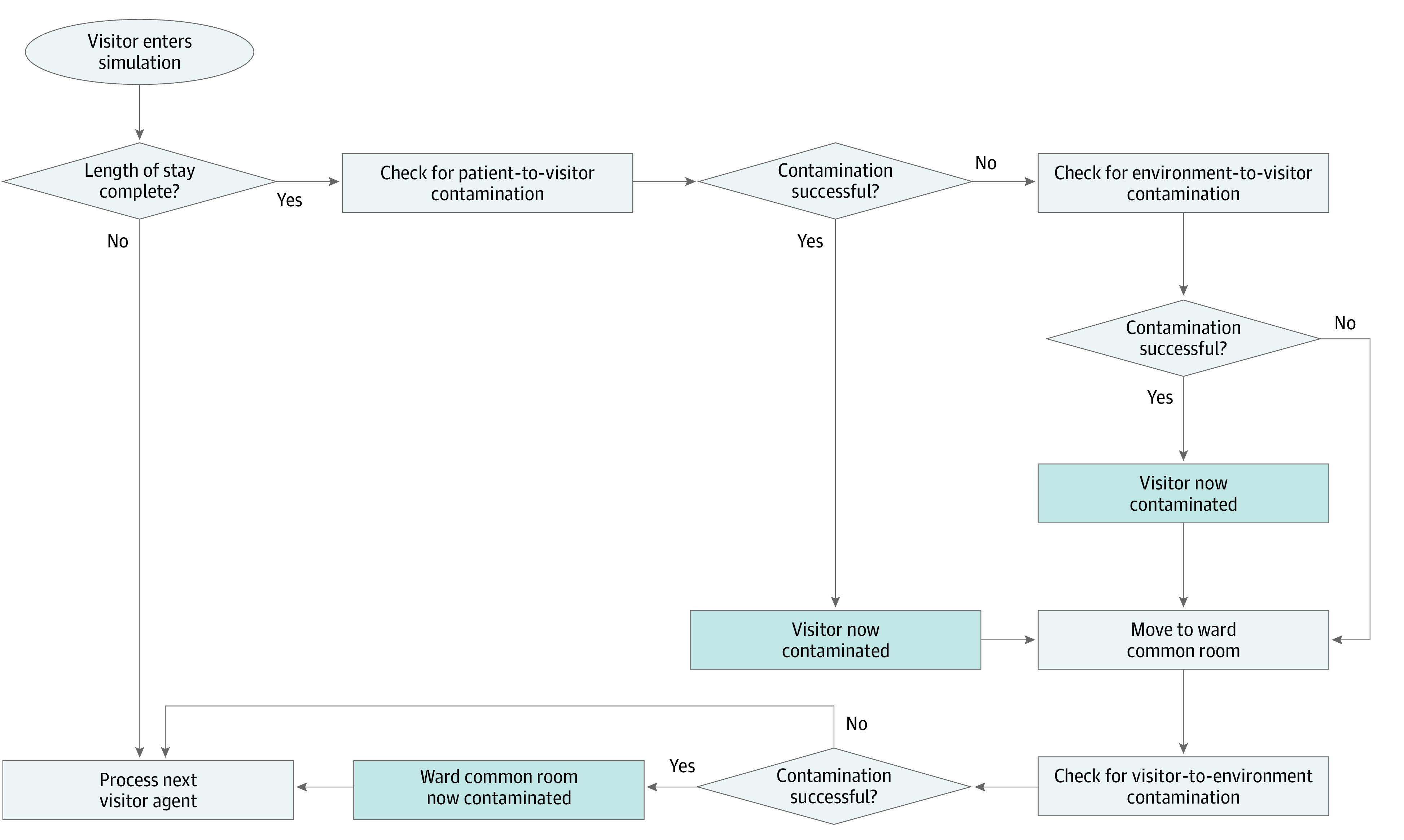
Logic of Visitor Agent Flow in the Agent-Based Model Pathways of visitors’ *Clostridiodides difficile* propagation based on visitor behavior, as shown in Barker et al.^4^

On each day in the simulation, there was a probability that a patient received up to 2 visitors. Visitors did not interact with other patients or health care workers in the hospital. Visitors could become transiently exposed to and contagious with *C difficile* through interactions with the patient with whom they visited and with contaminated environments. The probability of visitor exposure to *C difficile* in a patient room increased with the visit duration and depended on the transfer efficiency between surfaces. Visitors could wash their hands after exiting from the patient room according to hand hygiene recommendations regardless of VCP use. Visitors exited the hospital through a ward common room, which they contaminated with probability dependent on their length of stay in the common room. The baseline model assumed that visitors remained in the common room for 5 minutes before exiting. Each ward common room may also have been used by patients staying within the same ward. Therefore, a patient may have become exposed to *C difficile* through a common area contaminated by a visitor. Environment-to-agent contamination was possible through high-touch surfaces, which represent a portion of the room. Common rooms may have been cleaned daily depending on compliance with the bleach cleaning intervention. We assumed that visitors were healthy adults who were not colonized or infected with *C difficile*. Therefore, visitors could not shed *C difficile* but could expose and contaminate the environment. Figure 2 shows the logic governing visitor actions in the simulation, and the eAppendix in the Supplement further describes visitor-agent logic.

### Simulated Interventions

The model included the following core infection control interventions: nurse and physician hand hygiene, nurse and physician contact precautions, daily environmental cleaning, terminal environmental cleaning, visitor hand hygiene, visitor contact precautions, patient hand hygiene, and *C difficile* surveillance screening at admission. All interventions were implemented at 3 increasing levels of compliance: baseline, enhanced, and ideal. The baseline level corresponded to typical compliance with minimal institutional support or, for the surveillance intervention, no asymptomatic screening at admission. Enhanced and ideal implementation represented increasing levels of institutional support. Assumptions of baseline, enhanced, and ideal compliance for the core infection-control interventions were derived from the studies cited in eTable 2 in the Supplement. Parameters representing high-risk antibiotic use in the hospital remained constant for all interventions and were only changed when explicitly examined by the experiments.

### Simulation Settings and Outcomes

We simulated *C difficile* transmission at a generic 200-bed hospital for 13 months, including a 1-month warmup period. Data were collected during the following 12 simulated months. The primary output was the HO-CDI rate, calculated as the number of HO-CDI diagnoses made per 10 000 patient-days. In accordance with the definition from the Centers for Disease Control and Prevention, a CDI was classified as hospital onset if it was detected more than 3 days after admission.^15^ All CDIs detected within 3 days of admission were classified as community onset.

### Simulation Scenarios

We conducted 10 sets of simulation experiments to test the value of VCPs in different hospital settings under a variety of infection control implementation strategies. These experiments tested parameter assumptions associated with community distribution of CDI and susceptibility to infection, compliance of health care workers with hand hygiene protocols, *C difficile* transmission from visitors, number of visitors in the hospital, compliance of visitors with hand hygiene protocols, compliance with other infection control interventions, the discrete-time Markov chain representing patients’ disease-state transitions, health care worker distribution and behavior, and timing of patient arrivals (Table 1). The 10 experiment sets also included 1 experiment defined a posteriori, which considered the “worst case” hospital using parameters from analogous experiments most conducive to infection transmission.

**Table 1.** Modeled Experiment Scenarios

Experiment	Description
**0: Base case**	
0	All parameters for the model were baseline; VCPs were deimplemented
**1: Patient composition: varied the proportion of susceptible, colonized, and infected patients at admission**	
1a	Greater proportion of susceptible population: proportion of nonsusceptible patients at admission was decreased from 60% to 0%
1b	Higher colonization rates: proportion of colonized patients at admission was increased from 6% to 12%
1c	Higher infection rates: proportion of infected patients at admission was increased from 0.3% to 0.6%
1d	Higher at-risk population: all changes in scenarios 1a to 1c were used simultaneously
1e	Lower colonization rates: proportion of colonized patients at admission was decreased from 6% to 3%
1f	Lower infection rates: proportion of infected patients at admission was decreased from 0.3% to 0.15%
**2: Health care workers’ hand hygiene: varied nurses’ and physicians’ compliance with hand hygiene when caring for patients with and without CDI **	
2a	Lower hand hygiene compliance: hand hygiene compliance was decreased for care of patients without CDI (nurses, 60% to 30%; physicians, 50% to 25%) and patients with CDI (nurses, 69% to 35%; physicians, 62% to 31%)
2b	Enhanced hand hygiene compliance: hand hygiene compliance was increased to reflect an enhanced level of intervention implementation for care of patients without CDI (nurses, 60% to 79%; physicians, 50% to 71%) and patients with CDI (nurses, 69% to 84%; physicians, 62% to 77%)
2c	Ideal hand hygiene compliance: hand hygiene compliance was increased to reflect the ideal level of intervention implementation for care of patients without CDI (nurses, 60% to 96%; physicians, 50% to 91%) and patients with CDI (nurses, 69% to 97%; physicians, 62% to 93%)
**3: Transmission from visitors: varied the rate at which visitors interacted with the environment**	
3a	Higher transmission from visitors: increased the rate of contact between visitors and the common-room environment from 0.179 to the patient-to-common-room-environment rate of 0.358
**4: Number of visitors in hospital: varied the proportion of patients with visitors, the mean number of visitors that a patient received, and the length of time** **that visitors spent in the hospital**	
4a	Higher number of visitors: increased the mean number of visitors that a patient received per day from 2 to 5
4b	Longer stay of visitors in patient rooms: increased the mean amount of time visitors stayed in patient rooms from 15 to 30 min
4c	Higher number of patients receiving visitors: increased the daily probability of receiving visitors from 0.5 to 1 for each patient
**5: Visitor hand hygiene experiments: varied visitor hand hygiene compliance with patients with CDI and without CDI **	
5a	Lower visitor hand hygiene: decreased visitor compliance with hand hygiene with patients without CDI (from 35% to 17.5%) and patients with CDI (from 50% to 25%)
5b	Enhanced visitor hand hygiene: increased visitor compliance with hand hygiene to reflect an enhanced level of intervention implementation with patients without CDI (from 35% to 50%) and patients with CDI (from 50% to 65%)
5c	Ideal visitor hand hygiene: increased visitor compliance with hand hygiene to reflect an ideal level of intervention implementation with patients without CDI (from 35% to 84%) and patients with CDI (from 50% to 88%)
**6: Intervention fidelity: varied compliance with 5 infection control practices, including daily cleaning, terminal cleaning, limiting the use of fluoroquinolones, limiting the use of high-risk antibiotics, and patient hand hygiene**	
6a	Lower daily cleaning: decreased the proportion of rooms of patients with CDI and common rooms cleaned daily with bleach from 46% to 23%
6b	Lower terminal cleaning: decreased the proportion of patient rooms cleaned with bleach after patient discharge from 47% to 24%
6c	Higher fluoroquinolone use: increased the proportion of patients receiving fluoroquinolones from 8% to 15%
6d	Higher high-risk antibiotic use: increased the proportion of patients receiving nonfluoroquinolone high-risk antibiotics from 13% to 23%
6e	Lower patient hand hygiene: decreased compliance with hand hygiene among patients with CDI (from 33% to 17%) and patients without CDI (from 48% to 24%)
**7: Patient disease transition probability matrix: varied the discrete-time Markov chain underpinning the model between 10 matrices that reflect heterogeneity in CDI progression in humans**^a^	
7a	Matrix 1
7b	Matrix 2
7c	Matrix 3
7d	Matrix 4
7e	Matrix 5
7f	Matrix 6
7g	Matrix 7 (same as base case)
7h	Matrix 8
7i	Matrix 9
7j	Matrix 10
**8: Distribution and behavior of health care workers: varied the number of health care workers, the mean time they spend with patients, and the number of contacts between patients and health care workers**	
8a	Increased nurse staffing: increased nurses per ward from 4 to 8
8b	Increased physician staffing: increased physicians per ward from 2 to 4
8c	Decreased nurse staffing: decreased nurses per ward from 4 to 3
8d	Decreased physician staffing: decreased physicians per ward from 2 to 1
8e	Increased nursing visits: increased nursing visits per patient from 5 to 10
8f	Increased physician visits: increased physician visits per patient from 1 to 2
8g	Decreased nursing visits: Decreased nursing visits per patient from 5 to 3
8h	Increased length of nurse visits: changed the mean amount of time nurses spend with each patient from 4.7 to 9.3 min
8i	Increased length of physician visits: changed the mean amount of time physicians spend with each patient from 10.8 to 21.5 min
8j	Decreased length of nurse visits: changed the mean amount of time nurses spend with each patient from 4.7 to 2.3 min
8k	Decreased length of physician visits: changed the mean amount of time physicians spend with each patient from 10.8 to 5.4 min
8l	Increased probability of patient and nurse contact: changed the probability of contact between patients and nurses from the baseline of 0.358 to 0.588 for 4.7 min
8m	Increased probability of patient and physician contact: changed the probability of contact between patients and physicians from 0.688 to 0.903 for 10.8 min
8n	Decreased probability of patient and nurse contact: changed the probability of contact between patients and nurses from 0.358 to 0.197 for 4.7 min
8o	Decreased probability of patient and physician contact: changed the probability of contact between patients and physicians from 0.688 to 0.441 for 10.8 min
**9: Patient arrival: experiments varied the daily rate at which patients were admitted to the hospital**	
9a	Increased patient arrival rate: increased patient arrivals from 26 to 52 per day
9b	Decreased patient arrival rate: decreased patient arrivals from 26 to 13 per day
**10: Worst-case scenario**	
10	Defined a posteriori, given the results of the preceding experiments, this experiment was designed to obtain the maximum possible change in HO-CDI rate associated with VCPs; this scenario included a higher at-risk population at admission (1d), lower hand-hygiene compliance among health care workers (2a), transmission from visitors to the environment modeled as high as from colonized patients (3), increased visitor length of stay (4b), an increased number of patients with visitors (4c), lower hand-hygiene compliance among visitors (5a), lower daily-cleaning compliance (6a), lower terminal-cleaning compliance (6b), higher fluoroquinolone use (6c), higher high-risk antibiotic use (6d), lower hand-hygiene compliance among patients (6e), Matrix 10 used as the underlying discrete-time Markov chain (7j), fewer total nurses (8c), increased number and length of nurse and physician visits (8e, 8f, 8h, 8i), increased probability of contact between patients and health care workers (8l, 8m), and increased rate of new patient arrivals (9a)

Abbreviations: CDI, *Clostridioides difficile* infection; HO, hospital onset; VCP, visitor contact precaution.

^a^ More details are given in Codella et al.^14^

Baseline assumptions for the experiments are provided in eTable 2 in the Supplement. Individual experiments are described in detail in Table 1. In all the experiments, we compared 2 scenarios to estimate the maximum possible association between VCPs and HO-CDI: VCPs deimplemented (ie, no use of VCPs) vs VCPs implemented at an ideal level of compliance (93.5%). All interventions that were not assessed in the simulation scenario were implemented at their baseline level.

We then estimated the minimum required improvement in compliance from baseline that the interventions of health care workers’ hand hygiene, daily cleaning, and terminal cleaning would need to achieve a similar or greater association with HO-CDI rate reduction compared with ideal implementation of the VCP intervention.

### Statistical Analysis

We ran 5000 replications for each scenario to obtain stable estimates. Means and 95% CIs were calculated for all experiments. We used the SciPy Stats package (Enthought, Inc) to build 95% CIs with the Python programming language.^16^ We considered the association of VCPs with HO-CDI to be negligible if the difference between the control and experimental means was less than 1%.

## Results

The overall baseline rate of HO-CDI when all model input parameters were set at baseline and VCPs were deimplemented was 7.94 per 10 000 patient-days (95% CI, 7.91-7.98 per 10 000 patient-days). This changed to 7.97 per 10 000 patient-days (95% CI, 7.93-8.01 per 10 000 patient-days) with VCPs implemented at the ideal level (Table 2). The association between VCPs and HO-CDI rates followed expected trends. For example, an increased proportion of susceptible patients (HO-CDI rate with VCPs deimplemented, 17.18 per 10 000 patient-days; 95% CI, 17.13-17.24 per 10 000 patient-days), lower hand-hygiene compliance among health care workers (HO-CDI rate with VCPs deimplemented, 14.43 per 10 000 patient-days; 95% CI, 14.38-14.48 per 10 000 patient-days), and lower environmental cleaning compliance (HO-CDI rate with VCPs deimplemented, 11.32 per 10 000 patient-days; 95% CI, 11.28-11.37 per 10 000 patient-days) were associated with higher estimated rates of HO-CDI. Across all experiments, VCPs were associated with a change of less than 1% in the rates of HO-CDI (Table 2). The experiments varying the proportion of susceptible, colonized, and infected patients at admission, which correspond to assumptions regarding the prevalence of CDI in the community, suggested that the association between VCPs and HO-CDI was negligible across all admission populations. The reduction in HO-CDI rates was less than 1% even in the worst-case scenario.

**Table 2.** Association of Deimplementation vs Implementation of VCPs With CDI Rates by Experiment

Experiment	Description	HO-CDI rate, per 10 000 patient-days (95% CI)		Absolute change, %
		VCPs deimplemented	VCPs ideal	
**0: Base case experiment**				
0	Base case	7.94 (7.91-7.98)	7.97 (7.93-8.01)	<1
**1: Patient composition experiments**				
1a	Higher susceptible population	17.18 (17.13-17.24)	17.19 (17.14-17.25)	<1
1b	Higher colonization rates at admission	9.36 (9.32-9.40)	9.37(9.33-9.42)	<1
1c	Higher infection rates at admission	7.97 (7.93-8.00)	7.98 (7.94-8.02)	<1
1d	Higher at-risk population at admission	18.35 (18.29-18.40)	18.36 (18.31-18.42)	<1
1e	Lower colonization rates at admission	6.78 (6.75-6.82)	6.79 (6.76-6.83)	<1
1f	Lower infection rates at admission	7.91 (7.88-7.95)	7.91 (7.87-7.95)	<1
**2: Health care worker hand hygiene experiments**				
2a	Lower hand hygiene among health care workers	14.43 (14.38-14.48)	14.43 (14.38-14.48)	<1
2b	Enhanced hand hygiene among health care workers	5.31 (5.28-5.34)	5.32 (5.29-5.35)	<1
2c	Ideal hand hygiene among health care workers	3.84 (3.81-3.87)	3.84 (3.81-3.86)	<1
**3: Transmission from visitors experiment**				
3.	Higher rate of transmission from visitors to environment	7.93 (7.90-7.97)	7.95 (7.92-7.99)	<1
**4: Visitors in hospital experiments**				
4a	Higher number of visitors	7.94 (7.90-7.98)	7.97 (7.93-8.01)	<1
4b	Visitors stay longer	7.93 (7.89-7.97)	7.93 (7.89-7.97)	<1
4c	More patients have visitors	7.97 (7.93-8.00)	7.94 (7.90-7.98)	<1
**5: Visitor hand hygiene experiments**				
5a	Lower hand hygiene among visitors	7.96 (7.92-7.99)	7.96 (7.92-8.00)	<1
5b	Enhanced hand hygiene among visitors	7.94 (7.90-7.98)	7.95 (7.92-7.99)	<1
5c	Ideal hand hygiene among visitors	7.96 (7.93-8.00)	7.97 (7.93-8.00)	<1
**6: Intervention fidelity**				
6a	Lower daily cleaning	11.32 (11.28-11.37)	11.34 (11.30-11.39)	<1
6b	Lower terminal cleaning	8.30 (8.27-8.34)	8.28 (8.25-8.32)	<1
6c	Higher fluoroquinolone use	8.43 (8.40-8.47)	8.43 (8.39-8.47)	<1
6d	Higher high-risk antibiotic use	8.83 (8.79-8.87)	8.85 (8.81-8.89)	<1
6e	Lower hand hygiene among patients	8.54 (8.50-8.58)	8.51 (8.47-8.55)	<1
**7: Patient disease transition probability matrix used**				
7a	Matrix 1	9.12 (9.07-9.16)	9.10 (9.06-9.14)	<1
7b	Matrix 2	6.75 (6.71-6.78)	6.76 (6.72-6.79)	<1
7c	Matrix 3	9.06 (9.02-9.10)	9.07 (9.03-9.11)	<1
7d	Matrix 4	7.95-(7.92-7.99)	7.95 (7.91-7.99)	<1
7e	Matrix 5	8.81 (8.77-8.85)	8.82 (8.78-8.86)	<1
7f	Matrix 6	6.76 (6.73-6.80)	6.77 (6.73-6.80)	<1
7g	Matrix 7	7.94 (7.91-7.98)	7.97 (7.93-8.01)	<1
7h	Matrix 8	5.55 (5.52-5.58)	5.56 (5.53-5.59)	<1
7i	Matrix 9	7.87 (7.83-7.91)	7.85 (7.82-7.89)	<1
7j	Matrix 10	9.15 (9.11-9.19)	9.15 (9.11-9.19)	<1
**8: Health care worker distribution and behavior**				
8a	Increased nurses	8.18 (8.14-8.22)	8.18 (8.14-8.22)	<1
8b	Increased physicians	7.54 (7.50-7.58)	7.53 (7.50-7.57)	<1
8c	Decreased nurses	7.53 (7.49-7.57)	7.50 (7.46-7.54)	<1
8d	Decreased physicians	7.98 (7.94-8.01)	7.99 (7.95-8.02)	<1
8e	Increased nurse visits	11.52 (11.47-11.56)	11.53 (11.48-11.57)	<1
8f	Increased physician visits	10.31 (10.26-10.35)	10.32 (10.28-10.36)	<1
8g	Decreased nurse visits	5.41 (5.37-5.44)	5.43 (5.40-5.46)	<1
8h	Increased length of nurse visits	10.46 (10.42-10.5)	10.45 (10.41-10.50)	<1
8i	Increased length of physician visits	8.60 (8.56-8.63)	8.57 (8.53-8.61)	<1
8j	Decreased length of nurse visits	5.25 (5.22-5.28)	5.25 (5.22-5.28)	<1
8k	Decreased length of physician visits	7.07 (7.03-7.10)	7.07 (7.03-7.10)	<1
8l	Increased probability of patient and nurse contact	16.55 (16.5-16.61)	16.57 (16.51-16.62)	<1
8m	Increased probability of patient and physician contact	9.48 (9.44-9.52)	9.49 (9.44-9.53)	<1
8n	Decreased probability of patient and nurse contact	16.21 (16.16-16.27)	16.21 (16.16-16.27)	<1
8o	Decreased probability of patient and physician contact	9.45 (9.41-9.49)	9.45 (9.41-9.49)	<1
**9: Patient arrival**				
9a	Increased patient arrival rate	8.22 (8.19-8.25)	8.22 (8.18-8.25)	<1
9b	Decreased patient arrival rate	5.86 (5.81-5.90)	5.87 (5.82-5.91)	<1
**10: Worst-case scenario**				
10	Conditions from experiments 1d, 2a, 3, 4b, 4c, 5a, 6a, 6b, 6c, 6d, 6e, 7j, 8c, 8e, 8f, 8h, 8i, 8l, 8m, 9a	128.76 (128.65-128.86)	128.79 (128.68-128.90)	<1

CDI, *Clostridioides difficile* infection; HO, hospital onset; VCP, visitor contact precaution.

The experiments with the largest changes in the baseline HO-CDI rate were associated with patient disease states (experiment set 1 in Table 2) and high-impact interventions, such as hand hygiene of health care workers (experiment 2a) and daily cleaning (experiment 6a). Experiments sets 4 and 5, which changed the distribution and behavior of patients in the simulation, were associated with a change of <1% in the overall baseline HO-CDI rate of 7.94 per 10 000 patient days.

**Figure 3.**
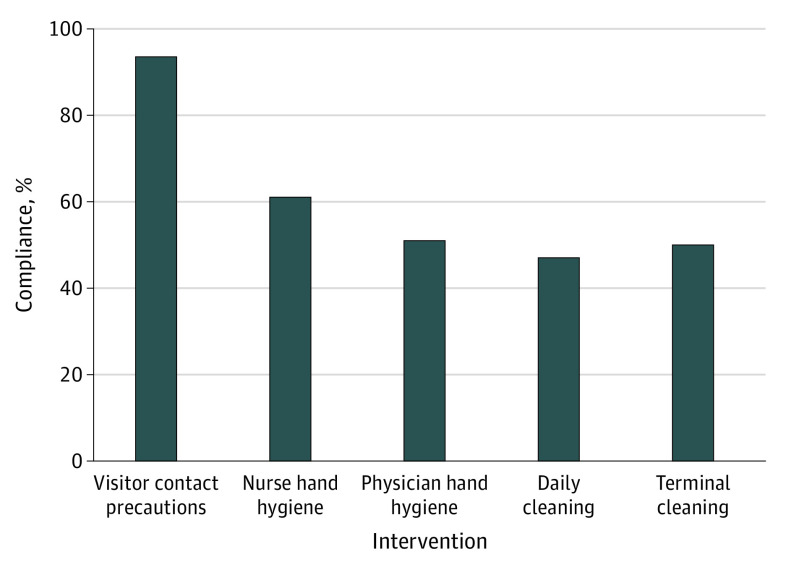
Compliance Associated With at Least 1% Decrease in Hospital-Onset *Clostridioides difficile* Infections per 10 000 Patient-Days by Intervention

Figure 3 and eTable 8 in the Supplement summarize the results of incremental-improvement experiments that increased the mean compliance with hand hygiene among health care workers from a baseline estimate of 55% to 56% (60% to 61% for nurses and 50% to 51% for physicians) and compliance with environmental cleaning from 47% to 49% (46% to 47% for daily cleaning and 47% to 50% for terminal cleaning). For health care workers’ hand hygiene and environmental cleaning, improvements in compliance of 3% or less were associated with greater CDI reduction compared with ideal VCP implementation.

## Discussion

To our knowledge, this is the first study to estimate the association of VCPs with HO-CDI rates. This simulation study suggests that VCPs have a minimal association with the overall HO-CDI rate and that similar benefits could be obtained by increasing compliance with core infection-control interventions. such as health care workers’ hand hygiene or environmental cleaning. The VCPs were not associated with a great reduction in HO-CDI using simulation models with any of the examined hospital configurations or patient profiles. The adaptability of this model may enable these experiments to be run for different hospital configurations and may help estimate the association between VCPs and HO-CDI in specific settings.

Because no previous study, to our knowledge, has evaluated the association of VCPs with HO-CDI, direct comparison with previous studies was not possible. However, multiple studies^17,18,19,20,21^ have described the harms associated with contact precautions for patients. Previous studies reported increased delirium and depression associated with contact precautions.^18,19,20^ Patients under contact precautions also reported lower care satisfaction.^21^ It is likely that these negative outcomes are associated with the use of VCPs.^17^

Other studies have questioned the effectiveness of VCPs for MRSA and VRE infections.^3,22,23^ The 2015 Society for Healthcare Epidemiology of America (SHEA) report^3^ states that VCPs are an ineffective intervention because of the high prevalence of MRSA and VRE in the community. As community spread is increasingly recognized in the context of *C difficile,* with some studies noting a general increase in community-onset CDI since the early 1990s, a similar phenomenon may explain the potential low impact of VCPs.^24^ The SHEA guidelines^3^ also suggest that visitor behavior, particularly that visitors normally do not interact with multiple patients, may be a reason why this intervention is associated with little reduction in rates of HO-CDI.

High fidelity and sustained implementation of VCPs require considerable resources. The SHEA guidelines^3^ report the difficulty of educating visitors and enforcing compliance with contact-precaution practices as reasons against the use of contact precautions among visitors of patients with MRSA and VRE infection. These reasons likely generalize to *C difficile* because the recommended gowns, gloves, and visitor education for MRSA and VRE infections are similar to those for CDI.

Our findings may have implications for health care institutions, infection prevention programs, and clinicians. The minimal changes to the baseline HO-CDI rate associated with VCPs suggest that any restrictions regarding visitor length of stay or number of visitors allowed may be relaxed with minimal change in HO-CDI rates. Removing VCPs for CDI control may also be associated with decreased health care worker burden related to enforcing and managing compliance with VCPs, allowing additional time and effort to be allocated toward more effective interventions.^25,26,27^ These interventions include hand hygiene of health care workers and environmental cleaning, which have consistently been associated with significant decreases in HO-CDI.^28,29,30^

The personal protective equipment (PPE) resources saved by removing VCPs can also be used to improve PPE inventory for use during acute infectious outbreaks. For example, in the coronavirus disease 2019 outbreak, guaranteeing PPE to health professionals became a challenge, with calls for removing contact precautions for patients with MRSA and VRE infection during the pandemic.^31,32^ Minimizing PPE use among visitors to rooms of patients with CDI would provide much-needed additional resources for health care workers at seemingly minimal risks for worsening *C difficile* transmission.

### Limitations

This study has limitations. First, all data analyzed in this study were generated through simulation and not through clinical experiments. Therefore, any conclusions drawn from this study should at most be used to influence the purpose or design of such clinical experiments. Second, we did not consider the effect of community-onset CDI on hospital visitors. The model assumed that visitors could not be infected or colonized, and thus, visitors were not able to expose patients to *C difficile* spores from the community. This study did not consider the benefit associated with VCPs among visitors. The model also simulated each ward uniformly and did not account for differential CDI risk across wards. We also did not consider how the use or deimplementation of VCPs may affect compliance with other infection control measures, such as hand hygiene or precautions for health care worker contact. It is possible that deimplementing VCPs would suggest to visitors and health care workers a diminished risk of *C difficile*, which in turn may be associated with decreased compliance with other infection-control practices that are highly effective against HO-CDI. A recent study of health care workers found that the use of contact precautions was associated with increased risk-mitigating behavior, such as improved hand hygiene.^33^ However, another study suggests that a threshold of patients designated under isolation exists at which compliance with contact precautions decreases.^34^ These studies suggest interactions between infection control interventions and their perceived importance; further work is required to understand whether such an association could be present after deimplementation of VCPs among patients with CDI. This study also did not examine the impact of VCPs in a pediatric setting, where visitor-patient interactions differ from the adult, acute care hospital setting. However, a previous agent-based model^35^ of *C difficile* transmission in a pediatric hospital showed little reduction in HO-CDI in association with implementation of VCPs at an ideal level under baseline assumptions. Further research is required to estimate the contribution of VCPs in a pediatric care setting under different configurations. In addition, the assumption that visitors can only contaminate the environment, not health care workers or other patients, may be a limitation. However, given that most visitors do not physically interact with more than 1 patient, it is unlikely that changing this assumption would greatly affect the current model outcomes.

## Conclusions

In this simulation study, VCPs were associated with minimal change in HO-CDI rates. In the context of these findings and financial and supply-chain constraints, the infection control community should reevaluate the value of VCPs for patients with CDI. If this model is correct, removing VCPs, which are associated with minimal change in HO-CDI rates, from prevention bundles may allow better allocation of resources for containment.

## Notes

Supplement. **eAppendix** **eTable 1.** Clinical states of the patients included in the discrete-time Markov chain **eFigure.** Possible transitions between CDI disease states **eTable 2.** Key input parameters **eTable 3.** Performance of infection control measures implemented at enhanced adherence, as quantified by NetLogo and Java simulation **eTable 4.** Performance of infection control measures implemented at ideal adherence, as quantified by NetLogo and Java simulation **eTable 5.** Stability of baseline infection outcomes as a function of number of replications, as quantified by NetLogo and Java models **eTable 6.** HO-CDI per 10,000 patient days as a function of compliance with surveillance, under maximal visitor transmission **eTable 7.** HO-CDI per 10,000 patient days with VCPs in use for all patients **eTable 8. **Changes in hand hygiene and cleaning compliance associated with reduction in HO-CDI/10,000 patient days on par with that of VCPs ideal intervention **eReferences**
