# Supplementary material for: Association of Visitor Contact Precautions With Estimated Hospital-Onset Clostridioides difficile Infection Rates in Acute Care Hospitals
Source: JAMA Netw Open. 2021 Feb 26;4(2):e210361. doi: 10.1001/jamanetworkopen.2021.0361 (PMC7910816; doi:10.1001/jamanetworkopen.2021.0361)
Supplement: Supplement. — eAppendix eTable 1. Clinical states of the patients included in the discrete-time Markov chain eFigure. Possible transitions between CDI disease states eTable 2. Key input parameters eTable 3. Performance of infection control measures implemented at enhanced adherence, as quantified by NetLogo and Java simulation eTable 4. Performance of infection control measures implemented at ideal adherence, as quantified by NetLogo and Java simulation eTable 5. Stability of baseline infection outcomes as a function of number of replications, as quantified by NetLogo and Java models eTable 6. HO-CDI per 10,000 patient days as a function of compliance with surveillance, under maximal visitor transmission eTable 7. HO-CDI per 10,000 patient days with VCPs in use for all patients eTable 8. Changes in hand hygiene and cleaning compliance associated with reduction in HO-CDI/10,000 patient days on par with that of VCPs ideal intervention eReferences [file jamanetwopen-e210361-s001.pdf]

Supplementary Online Content

Scaria E, Barker AK, Alagoz O, Safdar N. Association of visitor contact precautions with estimated hospital-onset *Clostridioides difficile* infection rates in acute care hospitals. *JAMA Netw Open*. 2021;4(2):e210361. doi:10.1001/jamanetworkopen.2021.0361

eAppendix

eTable 1. Clinical states of the patients included in the discrete-time Markov chain

eFigure. Possible transitions between CDI disease states

eTable 2. Key input parameters

eTable 3. Performance of infection control measures implemented at enhanced adherence, as quantified by NetLogo and Java simulation

eTable 4. Performance of infection control measures implemented at ideal adherence, as quantified by NetLogo and Java simulation

eTable 5. Stability of baseline infection outcomes as a function of number of replications, as quantified by NetLogo and Java models

eTable 6. HO-CDI per 10,000 patient days as a function of compliance with surveillance, under maximal visitor transmission

eTable 7. HO-CDI per 10,000 patient days with VCPs in use for all patients

eTable 8. Changes in hand hygiene and cleaning compliance associated with reduction in HO-CDI/10,000 patient days on par with that of VCPs ideal intervention

eReferences

This supplementary material has been provided by the authors to give readers additional information about their work.

© 2021 Scaria E et al. *JAMA Network Open*.

eAppendix

Representing patient disease state

Patient *Clostridioides difficile* infection (CDI) state is modeled using a discrete-time Markov chain containing nine states, as described in eTable 1. The states of death and non-susceptible are absorbing, from which patients cannot transition to any other state. Possible transitions between disease states are illustrated in the eFigure.

Input parameters for the baseline model configuration

All infection control interventions included in the agent-based simulation model can be implemented at multiple levels representing varying compliance with protocols. Interventions can be implemented at the baseline level, representing minimal (if any) institutional support, and therefore have the least agent compliance. Interventions can also be implemented at an ideal level, representing significant institutional support and agent compliance. The simulation model includes several parameters to describe the baseline state, distribution, and behavior of agents in the model. The parameters that were directly referenced or altered in our study of VCPs de-implementation are described in eTable 2. For a full description of the model's input parameters and their derivations, please see Barker, et al 2018.[1]

Detailed description of visitor behavior

Visitors may enter the simulation between the in-model hours of 9:00am and 9:00pm. When visitor agents are initialized, they are assigned a length of stay according to an exponential distribution with a mean of 15 minutes. While the visitor is in the patient room, healthcare workers may enter and interact with the patient and the environment, but not with the visitor. After the visitor has completed their stay, probabilities for patient-to-visitor and environment-to-visitor contamination trials are calculated, depending on the visitor length of stay, frequency of

contact, proportion of the room or patient that may harbor *C. difficile spores,* and the transfer efficiency between surfaces. The proportion of the room or patient harboring *C. difficile* depends on the patient's disease status. If either of these probability trials are successful, the visitor is considered contaminated.

Right before leaving the patient room, Bernoulli trials are used to determine if the patient washed their hands upon exit or was wearing VCPs during their stay. These trials are dependent on the compliance and effectiveness of the two interventions. If either trial is successful, the visitor is no longer considered contaminated. The visitor moves to the ward common ward, where they remain for five minutes. The model then calculates the probability for a visitor-to-environment contamination trial, depending on visitor length of stay, frequency of contact, and transfer efficiency between surfaces. If the trial is successful and the visitor was contaminated, high touch surfaces in the common room are considered contaminated. The visitor is then removed from the model.

Conversion of model from NetLogo to Java

The NetLogo model described in Barker, et al simulates the spread of hospital onset *Clostridioides difficile* infection (HO-CDI) in a generic, 200-bed acute care hospital.[1] Though NetLogo's ease of use and graphical interface are highly useful features in modeling infectious diseases, other programming languages can offer greater flexibility and speed. Java is one such language and lends itself naturally to agent-based modeling because of its object-oriented capabilities. To enable the experiments described in this paper we first converted the model described in Barker et al to an analogous model in Java (version 8); see the Appendix material of Barker et al for detailed logic governing agent and environment interactions.[1]

© 2021 Scaria E et al. *JAMA Network Open*.

Coding and debugging of the Java model was completed in Eclipse IDE v4.8 (Photon), developed by the Eclipse Foundation. The Java model replicates the agent and environmental variables and behavior, with changes made as necessary to accommodate Java data structures and objects. Both the NetLogo and Java models use the Colt Project's Mersenne Twister algorithm to produce random number generator streams. ETables 3 and 4 compare the output of the NetLogo and Java models for different infection control measures implemented at the enhanced and ideal levels, respectively. Of primary interest was the percent change in HO-CDI per 10000 patient days and colonizations per 1000 admitted patients as a function of different infection control implementation. Absolute percent changes of less than one percent were considered insignificant. Like the NetLogo model, the Java model was face validated by subject matter experts in hospital associated infections and simulation.

To determine stability of results, HO-CDI per 10000 patient days and colonizations per 1000 admitted patients were examined as a function of number of replications, as seen in eTable 5. As the Java model showed a similar stability at 5000 replications to the NetLogo model, we concluded that 5000 replications were sufficient to produce stable results.

Existence of association between VCPs and HO-CDI reduction

In the interest of finding what conditions exist in our model, if any, where VCPs are associated with a greater than 1% reduction in HO-CDI rates, we conducted further experiments using large parameter changes. Of primary interest is if there exists a threshold at of visitor length of stay or rate of contact at which VCPs are associated with HO-CDI reductions. In our model, all probabilities related to visitor *C. difficile* acquisition or deposition are of the form:

$$p = 1 - DRe^{-l\lambda}$$

© 2021 Scaria E et al. *JAMA Network Open*.

Where:

$$p = \text{probability of acquisition or deposition}$$
$$D = \text{proportion of environment/patient contaminated with } C.\ difficile$$
$$R = \text{transfer probability of } C.\ difficile \text{ between patient/environment and visitor}$$
$$\lambda = \text{ rate of contact between visitor and environment/patient}$$
$$l = \text{ length of stay with patient/in environment}$$

As visitor length of stay and visitor acquisition/deposition rates increase, the probability of transfer rapidly approaches 1. Once the exponential power is on the order of -10, increasing visitor length of stay or rate of contact more do little to affect the probability of exposure. Therefore, there is little need to investigate lengths of stay or rates of contact that result in a power less than -10. We conducted an additional experiment where we multiplied the rates of contact between visitors and the environment and between visitors and patients by 10, thereby bringing the probability of transmission and deposition near to 1. The rate of HO-CDI per 10,000 patient days with no VCPs was 7.95 (95% CI: [7.91, 7.99]), while the rate with ideal VCPs implementation was 7.99 (95% CI: [7.95, 8.03]). As the difference between these average rates is less than one percent, we are confident that increasing the rate of contact or length of stay any further would not lead to a greater association between VCPs and HO-CDI.

We believe it would be of interest to know if there is any condition where VCPs may be associated with a larger reduction in HO-CDI. We found that under extreme conditions where the only possible transmission pathway was through visitors contaminating the common room, transmission to and from visitors was guaranteed, and surveillance testing was adequately high, VCPs could have a larger association with HO-CDI. Note that surveillance testing represents the scenario that patients are screened for *C. difficile* at admission and then are put in isolation therefore VCPs will be implemented. Experiments varying the surveillance testing parameter

© 2021 Scaria E et al. *JAMA Network Open*.

are shown in eTable 6. Averages and confidence intervals were calculated from 5000 replications.

Surveillance likely played an important role in the experiments described in eTable 6 because our model assumes that VCPs may only be used when interacting with patients known to be infected or colonized with *C. difficile.* Therefore, increasing surveillance and testing leads to a greater number of known colonizations and infections and thus increased VCPs use. However, even when all patients entering the hospital are tested (i.e., surveillance compliance of 100%), the association of VCPs and HO-CDI is still relatively small.

Under baseline conditions, simply expanding use of VCPs to all patients is not significantly associated with a decrease in HO-CDI, as described in eTable 7.

© 2021 Scaria E et al. *JAMA Network Open*.

eTable 1: Clinical states of the patients included in the discrete-time Markov chain (adapted from Barker et al 2018)[1]

**Clinical State**	**Description**
Non-susceptible	Not at risk for colonization or CDI
Susceptible	At risk for *C. difficile* colonization but no symptoms or disease
Exposed	Exposed to *C. difficile* but no symptoms or disease
Colonized	Gastrointestinal colonization of *C. difficile, but n*o symptoms
Infected	Clinically diagnosed CDI, symptomatic
Cleared	Experienced infection or colonization but successfully cleared
Recolonized	Recovered from infection, but gastrointestinal colonization remains, no symptoms
Infection recurrence	Recurrence of the infection in a previously infected patient, symptomatic
Death	CDI-related death

Patients in the clinical states that are shaded with gray are contagious, therefore can transmit *C. difficile* to other agents and contaminate the environment, while patients in other clinical states cannot

© 2021 Scaria E et al. *JAMA Network Open*.

eFigure: Possible transitions between CDI disease states (adapted from Barker et al 2018)[1]

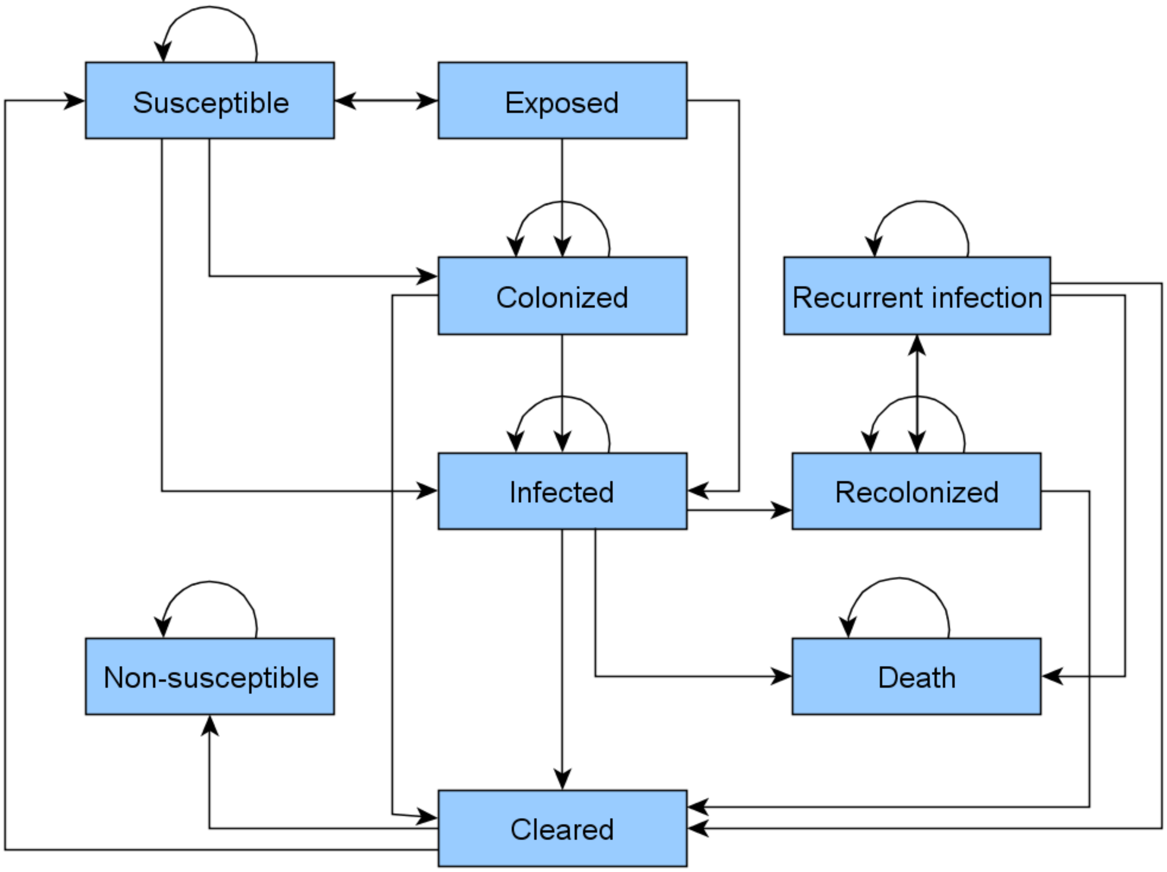

© 2021 Scaria E et al. *JAMA Network Open*.

eTable 2: Key Input Parameters (adapted from Barker et al 2018)[1]

Interventions Parameters				
**Intervention**	**Baseline Compliance**	**Ideal Compliance**	**Source**	**Source Dates**
Hand Hygiene				
Standard Patient				
Patient	33%	84%	2–7	04/03 - 2014
Nurse	60%	96%	8–18	08/2000-2012
Doctor	50%	91%	8–20	2000-12/12
Visitor	35%	84%	5,9,21–26	06/97 - 2016
CDI Patient				
Patient	48%	88%	27–30	10/02-12/12
Nurse	69%	97%	27–30	10/02-12/12
Doctor	61%	93%	27–30	10/02-12/12
Visitor	50%	88%	27–30	10/02-12/12
Environmental cleaning				
Daily Cleaning	46%	94%	31–35	03/01-05/12
Terminal cleaning	47%	98%	31,36–39	10/11-07/14

Admission Parameters			
**Parameter**	**Mean value**	**Source**	**Source Dates**
Proportion of susceptible patients	39.70%	40–44	01/99-12/12
Proportion of asymptomatic colonized patients	6.10%	45–55	03/06-03/15
Proportion of patients with CDI	0.29%	45,52,56,57	01/09-2016
Proportion of non-susceptible patients	53.90%	-	
Patient arrival rate	26/day	58,59	01/75-12/15

Health care worker behavior and distribution			
**Parameter**	**Value**	**Source**	**Source Dates**
Patient-nurse contact probability [rate]	0.358 (for 5 minutes) [10.53 contacts/ minute]	60	06/10-08/10
Patient-doctor contact probability [rate]	0.688 (for 5 minutes) [9.25 contacts/ minute]	60	06/10-08/10
Number of nurses per ward	4	60–63	04/98-08/10
Number of doctors per ward	2	58,60	06/10-12/12
Average nurse service time	4.7 minutes	28,60,64,65	06/01-04/13
Average doctor service time	10.8 minutes	28,60,64,65	06/01-04/13
Average number of nurse visits per 6 hours	5	28,60,66,67	06/01-04/13
Average number of doctor visits per 6 hours	1	28,60,66,67	06/01-04/13
Proportion of patients prescribed fluoroquinolines	7.5%	68–70	01/06-09/11
Proportion of patients prescribed other (non-fluoroquinolone) high risk antibiotics	12.5%	68–70	01/06-09/11

Visitor behavior			
**Parameter**	**Mean value**	**Source**	**Source Dates**
Probability of receiving visitors (per day)	0.5	71,72	06/72-03/04
Number of visitors per visit	2	72,73	2003-03/04
Visitor length of stay	15 minutes	60,72–74	2003-08/10

© 2021 Scaria E et al. *JAMA Network Open.*

Visitor-environment contact probability [rate]	0.932 (for 15 minutes) [0.179 contacts/ minute]	60	06/10-08/10

© 2021 Scaria E et al. *JAMA Network Open*.

eTable 3: Performance of infection control measures implemented at enhanced adherence, as quantified by NetLogo and Java simulation

Experiment		NetLogo, 5000 replications (95% CI)	Java, 5000 replications (95% CI)	Percent change relative to baseline, NetLogo	Percent change relative to baseline, Java
Baseline	HO-CDI per 10000 patient days	7.98 (7.95, 8.02)	7.98 (7.94, 8.01)	-	-
	Colonizations per 1000 patients	32.51 (32.45, 32.57)	31.90 (31.84, 31.97)	-	-
Daily cleaning enhanced	HO-CDI per 10000 patient days	2.48 (2.46, 2.50)	2.49 (2.47, 2.51)	68.92%	68.81%
	Colonizations per 1000 patients	7.32 (7.3, 7.35)	7.20 (7.18, 7.23)	77.48%	77.42%
Terminal cleaning enhanced	HO-CDI per 10000 patient days	6.52 (6.48, 6.55)	6.45 (6.42, 6.49)	18.30%	19.11%
	Colonizations per 1000 patients	23.57 (23.51, 23.62)	23.16 (23.11, 23.22)	27.50%	27.40%
Healthcare work hand hygiene enhanced	HO-CDI per 10000 patient days	5.37 (5.34, 5.40)	5.31 (5.28, 5.34)	32.71%	33.43%
	Colonizations per 1000 patients	20.26 (20.21, 20.31)	19.82 (19.77, 19.87)	37.68%	37.87%
Patient hand hygiene enhanced	HO-CDI per 10000 patient days	7.09 (7.05, 7.12)	7.04 (7.00, 7.07)	11.15%	11.80%
	Colonizations per 1000 patients	28.19 (28.14, 28.25)	27.66 (27.6, 27.71)	13.29%	13.31%
Visitor hand hygiene enhanced	HO-CDI per 10000 patient days	7.98 (7.94, 8.02)	7.96 (7.93, 8.00)	0.00%	0.15%
	Colonizations per 1000 patients	32.53 (32.47, 32.59)	31.88 (31.82, 31.94)	-0.06%	0.07%
Visitor contact precautions enhanced	HO-CDI per 10000 patient days	7.97 (7.93, 8.00)	7.96 (7.92, 8.00)	0.13%	0.17%
	Colonizations per 1000 patients	32.51 (32.45, 32.57)	31.90 (31.83, 31.96)	0.00%	0.03%
Healthcare worker contact precautions enhanced	HO-CDI per 10000 patient days	7.89 (7.86, 7.93)	7.84 (7.80, 7.87)	1.13%	1.75%
	Colonizations per 1000 patients	32.07 (32.01, 32.13)	31.45 (31.39, 31.51)	1.35%	1.43%
Patient transfer enhanced	HO-CDI per 10000 patient days	7.64 (7.60, 7.67)	7.60 (7.57, 7.64)	4.26%	4.70%
	Colonizations per 1000 patients	30.34 (30.28, 30.4)	29.75 (29.68, 29.81)	6.67%	6.77%
Surveillance enhanced	HO-CDI per 10000 patient days	5.13 (5.10, 5.16)	5.09 (5.06, 5.13)	35.71%	36.14%
	Colonizations per 1000 patients	19.78 (19.72, 19.83)	19.44 (19.38, 19.50)	39.16%	39.06%

© 2021 Scaria E et al. *JAMA Network Open*.

eTable 4: Performance of infection control measures implemented at ideal adherence, as quantified by NetLogo and Java simulation

Experiment		NetLogo, 5000 replications (95% CI)	Java, 5000 replications (95% CI)	Percent change relative to baseline, NetLogo	Percent change relative to baseline, Java
Baseline	HO-CDI per 10000 patient days	7.98 (7.95, 8.02)	7.98 (7.94, 8.01)	-	-
	Colonizations per 1000 patients	32.51 (32.45, 32.57)	31.9 (31.84, 31.97)	-	-
Daily cleaning ideal	HO-CDI per 10000 patient days	2.14 (2.12, 2.16)	2.16 (2.14, 2.18)	73.19%	72.92%
	Colonizations per 1000 patients	5.81 (5.79, 5.84)	5.70 (5.68, 5.73)	82.12%	82.13%
Terminal cleaning ideal	HO-CDI per 10000 patient days	6.08 (6.05, 6.11)	6.06 (6.02, 6.09)	23.84%	24.09%
	Colonizations per 1000 patients	21.23 (21.18, 21.28)	20.87 (20.82, 20.92)	34.69%	34.59%
Healthcare work hand hygiene ideal	HO-CDI per 10000 patient days	3.87 (3.84, 3.89)	3.84 (3.81, 3.86)	51.59%	51.91%
	Colonizations per 1000 patients	13.53 (13.49, 13.57)	13.23 (13.19, 13.27)	58.39%	58.55%
Patient hand hygiene ideal	HO-CDI per 10000 patient days	6.23 (6.20, 6.27)	6.20 (6.17, 6.24)	21.94%	22.24%
	Colonizations per 1000 patients	24.16 (24.11, 24.21)	23.67 (23.62, 23.73)	25.69%	25.80%
Visitor hand hygiene ideal	HO-CDI per 10000 patient days	8.00 (7.96, 8.03)	7.94 (7.91, 7.98)	-0.14%	0.40%
	Colonizations per 1000 patients	32.51 (32.45, 32.57)	31.90 (31.83, 31.96)	0.01%	0.02%
Visitor contact precautions ideal	HO-CDI per 10000 patient days	7.99 (7.96, 8.03)	7.97 (7.93, 8.01)	-0.12%	0.11%
	Colonizations per 1000 patients	32.52 (32.46, 32.58)	31.88 (31.81, 31.94)	-0.03%	0.09%

© 2021 Scaria E et al. *JAMA Network Open.*

Experiment		NetLogo, 5000 replications (95% CI)	Java, 5000 replications (95% CI)	Percent change relative to baseline, NetLogo	Percent change relative to baseline, Java
Healthcare worker contact precautions ideal	HO-CDI per 10000 patient days	7.80 (7.76, 7.84)	7.79 (7.76, 7.83)	2.29%	2.31%
	Colonizations per 1000 patients	31.78 (31.72, 31.84)	31.16 (31.1, 31.22)	2.25%	2.33%
Patient transfer ideal	HO-CDI per 10000 patient days	7.47 (7.44, 7.51)	7.47 (7.44, 7.51)	6.40%	6.32%
	Colonizations per 1000 patients	29.39 (29.34, 29.45)	28.85 (28.79, 28.91)	9.58%	9.58%
Surveillance ideal	HO-CDI per 10000 patient days	5.06 (5.03, 5.09)	5.02 (4.98, 5.05)	36.62%	37.12%
	Colonizations per 1000 patients	19.43 (19.38, 19.49)	19.08 (19.02, 19.14)	40.22%	40.19%

© 2021 Scaria E et al. *JAMA Network Open*.

eTable 5: Stability of baseline infection outcomes as a function of number of replications, as quantified by NetLogo and Java models

Model		Number of Replications				
		1000	2000	3000	4000	5000
NetLogo	HO-CDI per 10000 patient days	8.12 (8.03, 8.21)	8.06 (7.99, 8.12)	8.01 (7.96, 8.06)	8.01 (7.96, 8.05)	7.98 (7.95, 8.02)
	Colonizations per 1000 patients	32.43 (32.3, 32.57)	32.43 (32.3, 32.57)	32.47 (32.39, 32.55)	32.51 (32.44, 32.57)	32.51 (32.45, 32.58)
Java	HO-CDI per 10000 patient days	7.89 (7.81, 7.98)	7.96 (7.90, 8.02)	7.98 (7.93, 8.03)	7.98 (7.94, 8.02)	7.98 (7.95, 8.02)
	Colonizations per 1000 patients	31.79 (31.66, 31.93)	31.89 (31.79, 31.98)	31.93 (31.85, 32.01)	31.90 (31.83, 31.97)	32.51 (32.45, 32.57)

© 2021 Scaria E et al. *JAMA Network Open*.

eTable 6: HO-CDI per 10,000 patient days as a function of compliance with surveillance, under maximal visitor transmission

Compliance with surveillance	VCPs de-implemented HO-CDI/ 10,000 patient days (95% CI)	VCPs Ideal HO-CDI/ 10,000 patient days (95% CI)	Change
50%	30.03 (29.96, 30.1)	29.77 (29.7, 29.84)	0.89%
60%	29.83 (29.76, 29.9)	29.54 (29.47, 29.62)	0.97%
70%	29.7 (29.63, 29.78)	29.47 (29.4, 29.54)	0.80%
80%	29.59 (29.52, 29.66)	29.17 (29.1, 29.24)	1.42%
90%	29.46 (29.39, 29.53)	28.92 (28.85, 28.99)	1.85%
100%	29.29 (29.22, 29.36)	28.63 (28.56, 28.7)	2.25%

© 2021 Scaria E et al. *JAMA Network Open*.

eTable 7: HO-CDI per 10,000 patient days with VCPs in use for all patients

Description	VCPs de-implemented HO-CDI/ 10,000 patient days (95% CI)	VCPs Ideal HO-CDI/ 10,000 patient days (95% CI)	Change
Baseline conditions, VCPs available for use with all patients	7.95 (7.91, 7.98)	7.95 (7.91, 7.99)	<1%

© 2021 Scaria E et al. *JAMA Network Open*.

eTable 8: Changes in hand hygiene and cleaning compliance associated with reduction in HO-CDI/10,000 patient days on par with that of VCPs ideal intervention

Intervention	Baseline (no VCP use)		Improved		Absolute percent reduction in HO-CDI rate
	Compliance (%)	HO-CDI/10,000 Patient days	Compliance (%)	HO-CDI/10,000 Patient days	
Visitor contact precautions	0	7.94 (7.91, 7.98)	93.5	7.97 (7.93, 8.01)	< 1%
Nurse hand hygiene	60	7.94 (7.91, 7.98)	61	7.82 (7.78, 7.86)	2.01%
Physician hand hygiene	50	7.94 (7.91, 7.98)	51	7.90 (7.86, 7.94)	1.00%
Daily cleaning	46	7.94 (7.91, 7.98)	47	7.86 (7.82, 7.90)	1.50%
Terminal cleaning	47	7.94 (7.91, 7.98)	50	7.88 (7.84, 7.92)	1.25%

© 2021 Scaria E et al. *JAMA Network Open*.

eReferences

1. Barker AK, Alagoz O, Safdar N. Interventions to Reduce the Incidence of Hospital-Onset Clostridium difficile Infection: An Agent-Based Modeling Approach to Evaluate Clinical Effectiveness in Adult Acute Care Hospitals. *Clin Infect Dis*. 2018;66(8):1192-1203. doi:10.1093/cid/cix962

2. Davis C. Infection-free surgery: how to improve hand-hygiene compliance and eradicate methicillin-resistant Staphylococcus aureus from surgical wards. *Ann R Coll Surg Engl*. 2010;92(4):316-319. doi:10.1308/003588410X12628812459931

3. Srigley JA, Furness CD, Gardam M. Measurement of Patient Hand Hygiene in Multiorgan Transplant Units Using a Novel Technology: An Observational Study. *Infect Control Hosp Epidemiol*. 2014;35(11):1336-1341. doi:10.1086/678419

4. Cheng VCC, Wu AKL, Cheung CHY, et al. Outbreak of human metapneumovirus infection in psychiatric inpatients: implications for directly observed use of alcohol hand rub in prevention of nosocomial outbreaks. *J Hosp Infect*. 2007;67(4):336-343. doi:10.1016/j.jhin.2007.09.010

5. Randle J, Arthur A, Vaughan N, Wharrad H, Windle R. An observational study of hand hygiene adherence following the introduction of an education intervention. *J Infect Prev*. 2014;15(4):142-147. doi:10.1177/1757177414531057

6. Hedin G, Blomkvist A, Janson M, Lindblom A. Occurrence of potentially pathogenic bacteria on the hands of hospital patients before and after the introduction of patient hand disinfection. *APMIS*. 2012;120(10):802-807. doi:10.1111/j.1600-0463.2012.02912.x

7. Gagné D, Bédard G, Maziade PJ. Systematic patients' hand disinfection: impact on meticillin-resistant Staphylococcus aureus infection rates in a community hospital. *J Hosp Infect*. 2010;75(4):269-272. doi:10.1016/j.jhin.2010.02.028

8. Dierssen-Sotos T, Brugos-Llamazares V, Robles-García M, et al. Evaluating the impact of a hand hygiene campaign on improving adherence. *Am J Infect Control*. 2010;38(3):240-243. doi:10.1016/j.ajic.2009.08.014

9. Randle J, Firth J, Vaughan N. An observational study of hand hygiene compliance in paediatric wards. *J Clin Nurs*. 2013;22(17/18):2586-2592. doi:10.1111/j.1365-2702.2012.04103.x

10. Monistrol O, Calbo E, Riera M, et al. Impact of a hand hygiene educational programme on hospital-acquired infections in medical wards. *Clin Microbiol Infect*. 2012;18(12):1212-1218. doi:10.1111/j.1469-0691.2011.03735.x

11. Tromp M, Huis A, de Guchteneire I, et al. The short-term and long-term effectiveness of a multidisciplinary hand hygiene improvement program. *Am J Infect Control*. 2012;40(8):732-736. doi:10.1016/j.ajic.2011.09.009

© 2021 Scaria E et al. *JAMA Network Open*.

12. Kowitt B, Jefferson J, Mermel LA. Factors associated with hand hygiene compliance at a tertiary care teaching hospital. *Infect Control Hosp Epidemiol*. 2013;34(11):1146-1152. doi:10.1086/673465

13. Mestre G, Berbel C, Tortajada P, et al. “The 3/3 Strategy”: A Successful Multifaceted Hospital Wide Hand Hygiene Intervention Based on WHO and Continuous Quality Improvement Methodology. *PLOS ONE*. 2012;7(10):e47200. doi:10.1371/journal.pone.0047200

14. Eldridge NE, Woods SS, Bonello RS, et al. Using the Six Sigma Process to Implement the Centers for Disease Control and Prevention Guideline for Hand Hygiene in 4 Intensive Care Units. *J Gen Intern Med*. 2006;21(Suppl 2):S35-S42. doi:10.1111/j.1525-1497.2006.00361.x

15. Zerr DM, Allpress AL, Heath J, Bornemann R, Bennett E. Decreasing hospital-associated rotavirus infection: a multidisciplinary hand hygiene campaign in a children’s hospital. *Pediatr Infect Dis J*. 2005;24(5):397-403.

16. Mayer J, Mooney B, Gundlapalli A, et al. Dissemination and sustainability of a hospital-wide hand hygiene program emphasizing positive reinforcement. *Infect Control Hosp Epidemiol*. 2011;32(1):59-66. doi:10.1086/657666

17. Muto CA, Blank MK, Marsh JW, et al. Control of an Outbreak of Infection with the Hypervirulent Clostridium difficile BI Strain in a University Hospital Using a Comprehensive “Bundle” Approach. *Clin Infect Dis*. 2007;45(10):1266-1273.

18. Grant AM, Hofmann DA. It’s Not All About Me: Motivating Hand Hygiene Among Health Care Professionals by Focusing on Patients. *Psychol Sci*. 2011;22(12):1494-1499. doi:10.1177/0956797611419172

19. Grayson ML, Russo PL, Cruickshank M, et al. Outcomes from the first 2 years of the Australian National Hand Hygiene Initiative. *Med J Aust*. 2011;195(10). Accessed May 9, 2017. https://www.mja.com.au/journal/2011/195/10/outcomes-first-2-years-australian-national-hand-hygiene-initiative

20. Pittet D, Simon A, Hugonnet S, Pessoa-Silva CL, Sauvan V, Perneger TV. Hand hygiene among physicians: performance, beliefs, and perceptions. *Ann Intern Med*. 2004;141(1):1-8.

21. Clock SA, Cohen B, Behta M, Ross B, Larson EL. Contact Precautions for Multidrug-Resistant Organisms (MDROs): Current Recommendations and Actual Practice. *Am J Infect Control*. 2010;38(2):105-111. doi:10.1016/j.ajic.2009.08.008

22. Birnbach DJ, Rosen LF, Fitzpatrick M, Arheart KL, Munoz-Price LS. An evaluation of hand hygiene in an intensive care unit: Are visitors a potential vector for pathogens? *J Infect Public Health*. 2015;8(6):570-574. doi:10.1016/j.jiph.2015.04.027

© 2021 Scaria E et al. *JAMA Network Open*.

23. Birnbach DJ, Nevo I, Barnes S, et al. Do hospital visitors wash their hands? Assessing the use of alcohol-based hand sanitizer in a hospital lobby. *Am J Infect Control*. 2012;40(4):340-343. doi:10.1016/j.ajic.2011.05.006

24. Caroe Aarestrup S, Moesgaard F, Schuldt-Jensen J. NUDGING HOSPITAL VISITORS' HAND HYGIENE COMPLIANCE. Published online 2016. http://inudgeyou.com/wp-content/uploads/2016/05/Hand_Hygiene.pdf

25. Nishimura S, Kagehira M, Kono F, Nishimura M, Taenaka N. Handwashing before entering the intensive care unit: What we learned from continuous video-camera surveillance. *Am J Infect Control*. 1999;27(4):367-369. doi:10.1016/S0196-6553(99)70058-1

26. Randle J, Arthur A, Vaughan N. Twenty-four-hour observational study of hospital hand hygiene compliance. *J Hosp Infect*. 2010;76(3):252-255. doi:10.1016/j.jhin.2010.06.027

27. Golan Y, Doron S, Griffith J, et al. The Impact of Gown-Use Requirement on Hand Hygiene Compliance. *Clin Infect Dis*. 2006;42(3):370-376. doi:10.1086/498906

28. Morgan DJ, Pineles L, Shardell M, et al. The effect of contact precautions on healthcare worker activity in acute care hospitals. *Infect Control Hosp Epidemiol*. 2013;34(1):69-73. doi:10.1086/668775

29. Swoboda SM, Earsing K, Strauss K, Lane S, Lipsett PA. Isolation status and voice prompts improve hand hygiene. *Am J Infect Control*. 2007;35(7):470-476. doi:10.1016/j.ajic.2006.09.009

30. Almaguer-Leyva M, Mendoza-Flores L, Medina-Torres AG, et al. Hand hygiene compliance in patients under contact precautions and in the general hospital population. *Am J Infect Control*. 2013;41(11):976-978. doi:10.1016/j.ajic.2013.05.003

31. Sitzlar B, Deshpande A, Fertelli D, Kundrapu S, Sethi AK, Donskey CJ. An environmental disinfection odyssey: evaluation of sequential interventions to improve disinfection of Clostridium difficile isolation rooms. *Infect Control Hosp Epidemiol*. 2013;34(5):459-465. doi:10.1086/670217

32. Goodman ER, Platt R, Bass R, Onderdonk AB, Yokoe DS, Huang SS. Impact of an Environmental Cleaning Intervention on the Presence of Methicillin-Resistant Staphylococcus aureus and Vancomycin-Resistant Enterococci on Surfaces in Intensive Care Unit Rooms. *Infect Control Hosp Epidemiol Off J Soc Hosp Epidemiol Am*. 2008;29(7):593-599. doi:10.1086/588566

33. Hayden MK, Bonten MJM, Blom DW, et al. Reduction in Acquisition of Vancomycin-Resistant Enterococcus after Enforcement of Routine Environmental Cleaning Measures. *Clin Infect Dis*. 2006;42(11):1552-1560. doi:10.1086/503845

34. Boyce JM, Havill NL, Dumigan DG, Golebiewski M, Balogun O, Rizvani R. Monitoring the effectiveness of hospital cleaning practices by use of an adenosine triphosphate

bioluminescence assay. *Infect Control Hosp Epidemiol*. 2009;30(7):678-684. doi:10.1086/598243

35. Hess AS, Shardell M, Johnson JK, et al. A randomized, controlled trial of enhanced cleaning to reduce contamination of healthcare worker gowns and gloves with multidrug-resistant bacteria. *Infect Control Hosp Epidemiol Off J Soc Hosp Epidemiol Am*. 2013;34(5):487-493. doi:10.1086/670205

36. Ramphal L, Suzuki S, McCracken IM, Addai A. Improving hospital staff compliance with environmental cleaning behavior. *Proc Bayl Univ Med Cent*. 2014;27(2):88-91.

37. Anderson DJ, Chen LF, Weber DJ, et al. Enhanced terminal room disinfection and acquisition and infection caused by multidrug-resistant organisms and Clostridium difficile (the Benefits of Enhanced Terminal Room Disinfection study): a cluster-randomised, multicentre, crossover study. *The Lancet*. 2017;0(0). doi:10.1016/S0140-6736(16)31588-4

38. Clifford R, Sparks M, Hosford E, et al. Correlating Cleaning Thoroughness with Effectiveness and Briefly Intervening to Affect Cleaning Outcomes: How Clean Is Cleaned? *PLoS ONE*. 2016;11(5). doi:10.1371/journal.pone.0155779

39. Carling PC, Parry MM, Rupp ME, et al. Improving cleaning of the environment surrounding patients in 36 acute care hospitals. *Infect Control Hosp Epidemiol*. 2008;29(11):1035-1041. doi:10.1086/591940

40. Agency for Healthcare Research and Quality. Healthcare Cost and Utilization Project Statistical Brief #180: Overview of Hospital stays in the United States, 2012. Published online 2012. https://www.hcup-us.ahrq.gov/reports/statbriefs/sb180-Hospitalizations-United-States-2012.pdf

41. Centers for Disease Control. Number, rate, and average length of stay for discharges from short-stay hospitals, by age, region, and sex: United States, 2010. Published online 2010. https://www.cdc.gov/nchs/data/nhds/1general/2010gen1_agesexalos.pdf

42. Hicks LA, Bartoces MG, Roberts RM, et al. US Outpatient Antibiotic Prescribing Variation According to Geography, Patient Population, and Provider Specialty in 2011. *Clin Infect Dis*. 2015;60(9):1308-1316. doi:10.1093/cid/civ076

43. Frenk SM, Kit BK, Lukacs SL, Hicks LA, Gu Q. Trends in the use of prescription antibiotics: NHANES 1999–2012. Accessed January 16, 2017. http://jac.oxfordjournals.org

44. Dantes R, Mu Y, Hicks LA, et al. Association Between Outpatient Antibiotic Prescribing Practices and Community-Associated Clostridium difficile Infection. *Open Forum Infect Dis*. 2015;2(3). doi:10.1093/ofid/ofv113

45. Koo HL, Van JN, Zhao M, et al. Real-Time Polymerase Chain Reaction Detection of Asymptomatic Clostridium difficile Colonization and Rising C. difficile–Associated Disease Rates. *Infect Control Hosp Epidemiol*. 2014;35(6):667-673. doi:10.1086/676433

© 2021 Scaria E et al. *JAMA Network Open*.

46. Alasmari F, Seiler SM, Hink T, Burnham C-AD, Dubberke ER. Prevalence and risk factors for asymptomatic clostridium difficile carriage. *Clin Infect Dis*. 2014;59(2):216-222. doi:10.1093/cid/ciu258

47. Leekha S, Aronhalt KC, Sloan LM, Patel R, Orenstein R. Asymptomatic Clostridium difficile colonization in a tertiary care hospital: Admission prevalence and risk factors. *Am J Infect Control*. 2013;41(5):390-393. doi:10.1016/j.ajic.2012.09.023

48. Loo VG, Bourgault A-M, Poirier L, et al. Host and Pathogen Factors for Clostridium difficile Infection and Colonization. *N Engl J Med*. 2011;365(18):1693-1703. doi:10.1056/NEJMoa1012413

49. Eyre DW, Griffiths D, Vaughan A, et al. Asymptomatic Clostridium difficile Colonisation and Onward Transmission. *PLOS ONE*. 2013;8(11):e78445. doi:10.1371/journal.pone.0078445

50. Longtin Y, Paquet-Bolduc B, Gilca R, et al. Effect of Detecting and Isolating Clostridium difficile Carriers at Hospital Admission on the Incidence of C difficile Infections: A Quasi-Experimental Controlled Study. *JAMA Intern Med*. 2016;176(6):796-804. doi:10.1001/jamainternmed.2016.0177

51. Nissle K, Kopf D, Rösler A. Asymptomatic and yet C. difficile-toxin positive? Prevalence and risk factors of carriers of toxigenic Clostridium difficile among geriatric in-patients. *BMC Geriatr*. 2016;16:185. doi:10.1186/s12877-016-0358-3

52. Kagan S, Wiener-Well Y, Ben-Chetrit E, et al. The risk for Clostridium difficile colitis during hospitalization in asymptomatic carriers. *J Hosp Infect*. doi:10.1016/j.jhin.2017.01.013

53. Gupta S, Mehta V, Herring T, et al. A Large Prospective North American Epidemiologic Study of Hospital-Associated Clostridium difficile Colonization & Infection. *Int Clostridium Difficile Symp Bled Slov*. 2012;Abstract 020.

54. Hung Y-P, Lin H-J, Wu T-C, et al. Risk Factors of Fecal Toxigenic or Non-Toxigenic Clostridium difficile Colonization: Impact of Toll-Like Receptor Polymorphisms and Prior Antibiotic Exposure. *PLOS ONE*. 2013;8(7):e69577. doi:10.1371/journal.pone.0069577

55. Dubberke ER, Burnham C-AD. Diagnosis of Clostridium difficile Infection: Treat the Patient, Not the Test. *JAMA Intern Med*. 2015;175(11):1801-1802. doi:10.1001/jamainternmed.2015.4607

56. Lucado J, Gould C, Elixhauser A. Clostridium Difficile Infections (CDI) in Hospital Stays, 2009: Statistical Brief #124. In: *Healthcare Cost and Utilization Project (HCUP) Statistical Briefs*. Agency for Healthcare Research and Quality (US); 2012. Accessed February 19, 2017. http://www.ncbi.nlm.nih.gov/books/NBK92613/

© 2021 Scaria E et al. *JAMA Network Open*.

57. Evans ME, Simbartl LA, Kralovic SM, Jain R, Roselle GA. Clostridium difficile Infections in Veterans Health Administration Acute Care Facilities. *Infect Control Hosp Epidemiol*. 2014;35(8):1037-1042. doi:10.1086/677151

58. American Hospital Association. AHA Hospital Statistics. Published online 2016.

59. Centers for Disease Control. Table 89. Hospitals, beds, and occupancy rates, by type of ownership and size of hospital: United States, selected years 1975–2013. Published online 2015. https://www.cdc.gov/nchs/data/hus/2015/089.pdf

60. Cohen B, Hyman S, Rosenberg L, Larson E. Frequency of Patient Contact with Health Care Personnel and Visitors: Implications for Infection Prevention. *Jt Comm J Qual Patient Saf Jt Comm Resour*. 2012;38(12):560-565.

61. California Code of Regulations. Nurse service staff 2003. 22 C.C.R., Sec. 70217. Published online July 1, 2003. https://govt.westlaw.com/calregs/Document/I8612C410941F11E29091E6B951DDF6CE?viewType=FullText&originationContext=documenttoc&transitionType=CategoryPageItem&contextData=(sc.Default&bhcp=1

62. Spetz J, Donaldson N, Aydin C, Brown DS. How Many Nurses per Patient? Measurements of Nurse Staffing in Health Services Research. *Health Serv Res*. 2008;43(5 Pt 1):1674-1692. doi:10.1111/j.1475-6773.2008.00850.x

63. Aiken LH, Clarke SP, Sloane DM, Sochalski J, Silber JH. Hospital Nurse Staffing and Patient Mortality, Nurse Burnout, and Job Dissatisfaction. *JAMA*. 2002;288(16):1987-1993. doi:10.1001/jama.288.16.1987

64. Evans HL, Shaffer MM, Hughes MG, et al. Contact isolation in surgical patients: A barrier to care? *Surgery*. 2003;134(2):180-188. doi:10.1067/msy.2003.222

65. Barker AK, Codella J, Ewers T, Dundon A, Alagoz O, Safdar N. Changes to physician and nurse time burdens when caring for patients under contact precautions. Am J Infect Control. 2017;45(5):542-543. doi:10.1016/j.ajic.2017.01.026

66. Rubin MA, Jones M, Leecaster M, et al. A Simulation-Based Assessment of Strategies to Control Clostridium Difficile Transmission and Infection. *PLoS ONE*. 2013;8(11). doi:10.1371/journal.pone.0080671

67. McArdle FI, Lee RJ, Gibb AP, Walsh TS. How much time is needed for hand hygiene in intensive care? A prospective trained observer study of rates of contact between healthcare workers and intensive care patients. *J Hosp Infect*. 2006;62(3):304-310. doi:10.1016/j.jhin.2005.09.019

68. Magill SS, Edwards JR, Beldavs ZG, et al. Prevalence of antimicrobial use in US acute care hospitals, May-September 2011. *JAMA*. 2014;312(14):1438-1446. doi:10.1001/jama.2014.12923

© 2021 Scaria E et al. *JAMA Network Open*.

69. Baggs J, Fridkin SK, Pollack LA, Srinivasan A, Jernigan JA. Estimating National Trends in Inpatient Antibiotic Use Among US Hospitals From 2006 to 2012. *JAMA Intern Med*. 2016;176(11):1639-1648. doi:10.1001/jamainternmed.2016.5651

70. Fridkin S, Baggs J, Fagan R, et al. Vital Signs: Improving Antibiotic Use Among Hospitalized Patients. Published online March 2014.

71. Cross KW, Turner RD. Factors affecting the visiting pattern of geriatric patients in a rural area. *Br J Prev Soc Med*. 1974;28(2):133-139.

72. Eriksson T, Bergbom I. Visits to intensive care unit patients--frequency, duration and impact on outcome. *Nurs Crit Care*. 2007;12(1):20-26. doi:10.1111/j.1478-5153.2006.00196.x

73. Gonzalez CE, Carroll DL, Elliott JS, Fitzgerald PA, Vallent HJ. Visiting preferences of patients in the intensive care unit and in a complex care medical unit. *Am J Crit Care Off Publ Am Assoc Crit-Care Nurses*. 2004;13(3):194-198.

74. Fumagalli S, Boncinelli L, Lo Nostro A, et al. Reduced cardiocirculatory complications with unrestrictive visiting policy in an intensive care unit: results from a pilot, randomized trial. *Circulation*. 2006;113(7):946-952. doi:10.1161/CIRCULATIONAHA.105.572537

© 2021 Scaria E et al. *JAMA Network Open*.
